# Global Trends and Hotspots in Research on Rehabilitation Robots: A Bibliometric Analysis From 2010 to 2020

**DOI:** 10.3389/fpubh.2021.806723

**Published:** 2022-01-11

**Authors:** Xiali Xue, Xinwei Yang, Zhongyi Deng, Huan Tu, Dezhi Kong, Ning Li, Fan Xu

**Affiliations:** ^1^Institute of Sports Medicine and Health, Chengdu Sport University, Chengdu, China; ^2^School of Sports Medicine and Health, Chengdu Sport University, Chengdu, China; ^3^School of Public Health, Chengdu Medical College, Chengdu, China

**Keywords:** machine learning, bibliometric analysis, CiteSpace, trend, artificial intelligence, rehabilitation robot

## Abstract

**Background:** In recent years, with the development of medical science and artificial intelligence, research on rehabilitation robots has gained more and more attention, for nearly 10 years in the Web of Science database by journal of rehabilitation robot-related research literature analysis, to parse and track rehabilitation robot research hotspot and front, and provide some guidance for future research.

**Methods:** This study employed computer retrieval of rehabilitation robot-related research published in the core data collection of the Web of Science database from 2010 to 2020, using CiteSpace 5.7 visualization software. The hotspots and frontiers of rehabilitation robot research are analyzed from the aspects of high-influence countries or regions, institutions, authors, high-frequency keywords, and emergent words.

**Results:** A total of 3,194 articles were included. In recent years, the research on rehabilitation robots has been continuously hot, and the annual publication of relevant literature has shown a trend of steady growth. The United States ranked first with 819 papers, and China ranked second with 603 papers. Northwestern University ranked first with 161 publications. R. Riener, a professor at the University of Zurich, Switzerland, ranked as the first author with 48 articles. The *Journal of Neural Engineering and Rehabilitation* has the most published research, with 211 publications. In the past 10 years, research has focused on intelligent control, task analysis, and the learning, performance, and reliability of rehabilitation robots to realize the natural and precise interaction between humans and machines. Research on neural rehabilitation robots, brain–computer interface, virtual reality, flexible wearables, task analysis, and exoskeletons has attracted more and more attention.

**Conclusions:** At present, the brain–computer interface, virtual reality, flexible wearables, task analysis, and exoskeleton rehabilitation robots are the research trends and hotspots. Future research should focus on the application of machine learning (ML), dimensionality reduction, and feature engineering technologies in the research and development of rehabilitation robots to improve the speed and accuracy of algorithms. To achieve wide application and commercialization, future rehabilitation robots should also develop toward mass production and low cost. We should pay attention to the functional needs of patients, strengthen multidisciplinary communication and cooperation, and promote rehabilitation robots to better serve the rehabilitation medical field.

## Introduction

In the era of the COVID-19 pandemic, reducing doctor–patient contact is an important measure to prevent crossinfection. The use of rehabilitation robots to assist healthcare workers in treating patients with COVID-19 has reduced doctor–patient contact and increased productivity. Rehabilitation robotics is a relatively young and rapidly growing field with increasing penetration into the clinical environment (1, 2). Functional rehabilitation and the auxiliary robot have gradually become important technical means of clinical rehabilitation treatment in the world and have spawned several new rehabilitation robot technologies and systems (3). They have received great attention from all countries and have developed with each passing day (4). Rehabilitation robots, originating from the research field of engineering, are the perfect combination of rehabilitation medicine and robot technology, which makes up for the deficiency of traditional rehabilitation treatment methods in ensuring the high intensity of rehabilitation training, the persistence of endurance, and the standardization of training effects. They integrate the knowledge of artificial intelligence, biomechanics, information science, and rehabilitation medicine and use intelligent bionic technology to assist patients to complete limb-training movements and achieve the purpose of rehabilitation. These robots are used mainly for the assistance and rehabilitation of the daily life of the disabled (5–8).

According to the functional classification, rehabilitation robots are mainly divided into three types: full-body rehabilitation robots (9), upper limb robots (10), and lower limb rehabilitation robots (11). According to the means of application, they can be divided into fixed wearable robots, such as exoskeleton robots. The exoskeleton robot is mainly used to assist walking and is a special type of rehabilitation robot. Stationary robots are mainly used for rehabilitation training. Exoskeletons and soft-bodied robots are divided according to how the driving force is transmitted (12–14). Functional rehabilitation robots are mainly applied to chronic patients with stroke, cerebral palsy, spinal cord injury, paraplegia, traumatic brain injury, and limb injury. Due to the limited function recovery effect of traditional rehabilitation training methods on chronic injury, rehabilitation robot-assisted training is being studied and applied more and more (15–20). At present, rehabilitation robots have been widely used in rehabilitation nursing, prosthesis, and rehabilitation therapy, which not only promotes the development of rehabilitation medicine but also drives the development of new technologies and new theories in related fields. At the same time, there are still many challenges in the development of rehabilitation robots, there are still controversies in the research on rehabilitation robots, such as safety problems, and the actual operation is not simple and convenient, so that it is limited to large hospitals; moreover, the number of people who are suitable and can use rehabilitation robots is small, and the actual utilization rate is low (21, 22). For various rehabilitation robots with different characteristics and advantages, the application effect of different rehabilitation programs is not clear, so it is particularly important to formulate the user guide of rehabilitation robots to guide the clinical application of rehabilitation robots.

CiteSpace visual analysis software is a Java application that generates cocitation networks based on reference citations to reveal the structure of a particular research area, enabling visual exploration through knowledge discovery in bibliographic databases. Through progressive knowledge domain visualization, the most cited and critical documents, areas of expertise within the knowledge domain, and the emergence of research topics can be intuitively plotted to detect and visualize trends and patterns in the scientific literature (23). The key issue in evidence-based medical practice is to find the best available evidence about a clinical problem, and the task can be simplified by combining visual analysis techniques with traditional methods developed in evidence-based medicine (24–27). After consulting the database, it was found that the research in the field of rehabilitation robots increased significantly after 2010. Therefore, this study took the literature related to rehabilitation robots collected in the core data collection of the Web of Science database from 2010 to 2020 as the research object (28). It uses CiteSpace 5.7 visualization software, combining the qualitative analysis of the online platform and the traditional method of literature metrology, research focus from the literature field, high-influence countries or regions, institutions, the author, the high-frequency keywords and mutation term, etc., on the international research hotspot in the field of rehabilitation robots and frontier parsing and tracking. The study also provides some references for the research on rehabilitation robots and the development of related subjects. At the same time, the future development trend of rehabilitation robots is prospected, which is of great significance to improve the function of rehabilitation robots and to develop and improve the future medical field.

## Methods

### Data Sources and Retrieval Strategies

The core data set of the Web of Science database was retrieved by computer, and the retrieval condition was “TS = (Rehabilitation Robot OR Rehabilitation Robotics).” The period is set as December 31, 2010 to December 31, 2020. The article is a peer-reviewed journal paper with good authority and representativeness. To ensure high-quality visualization results, the literature type in this study is limited to “article,” and the language type is set as “English.” It excludes conference abstracts, letters, reviews, news, journal reviews, and other literary types.

### Literature Screening

Two evaluators read the literature independently. First, preliminary screening was carried out according to the title and abstract of the article, and then screening was carried out again according to the inclusion and exclusion criteria. After the screening, if there was any dispute, the third evaluator read the whole paper, discussed it together, and decided whether or not to reject it.

### Data Analysis

CiteSpace 5.7 visualization software determines the information related to the literature according to the information of the first author in the article, mainly including the country, author, keywords, research institution, and so on. In the CiteSpace 5.7 visual software parameter setting, the period is set to 2010–2020; the time scale is set to 1 year; the threshold is set to “top N” and set to 50; and the top 50 representative papers are selected for systematic review and analysis. We chose Pathfinder as the clipping connection to simplify the network structure and highlight important features. Countries (regions), institutions, authors, cited authors, and keywords were selected for node type cooccurrence analysis to draw a visual atlas. CiteSpace was used to detect the mutation rate of keywords and generate a keyword ranking table with a high mutation rate. In the network graph, different nodes represent various elements, such as authors, institutions, countries, and keywords, whereas the size of nodes reflects the number or frequency of publications, and the connections between nodes represent relations such as cooperation, cooccurrence, or cocitation (23, 29–31).

## Results

### Bibliometric of Publication Output

**Figure 1:**
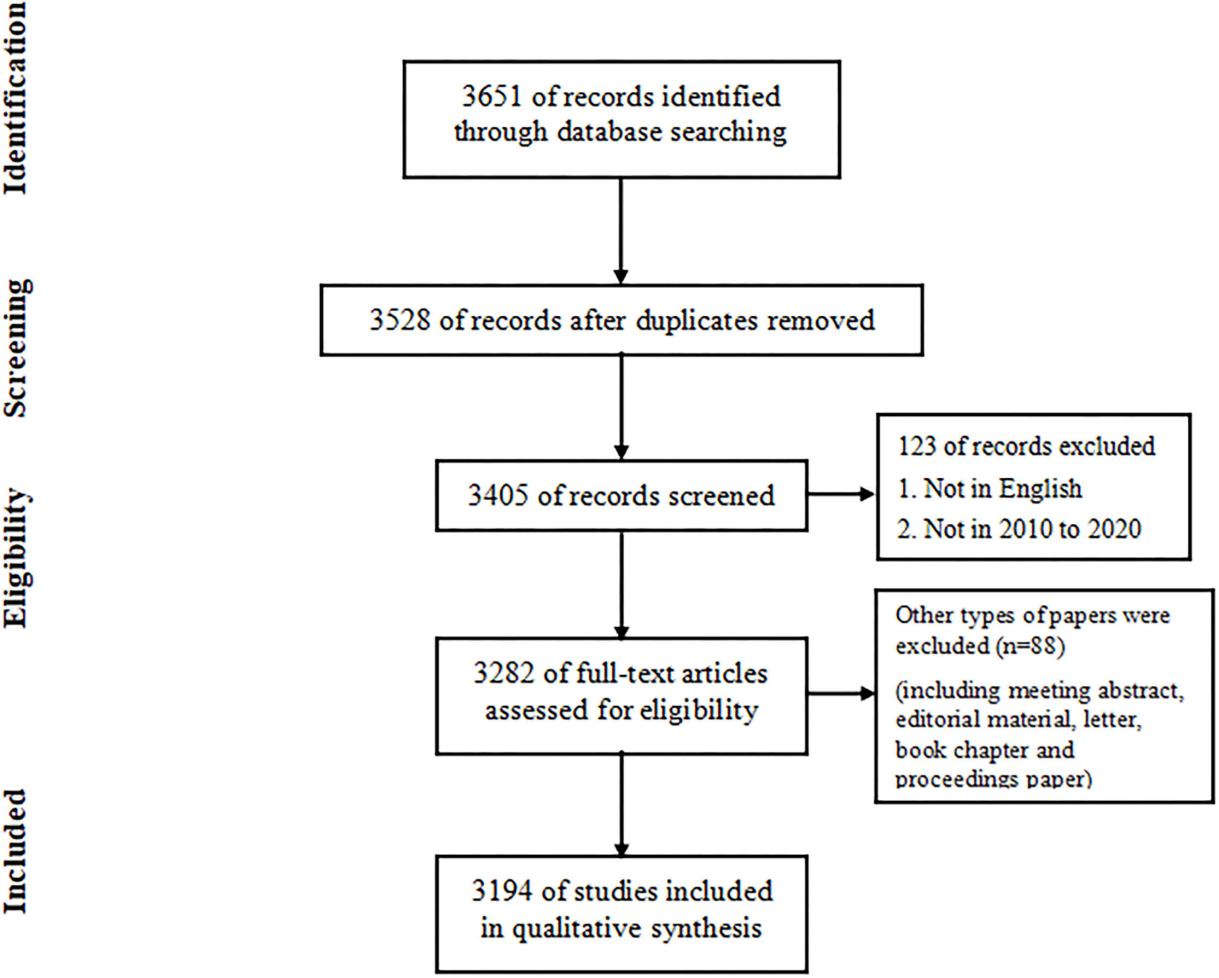
Flowchart of data filtration processing and excluding publications. WoSCC, Web of Science Core Collection.

**Figure 2:**
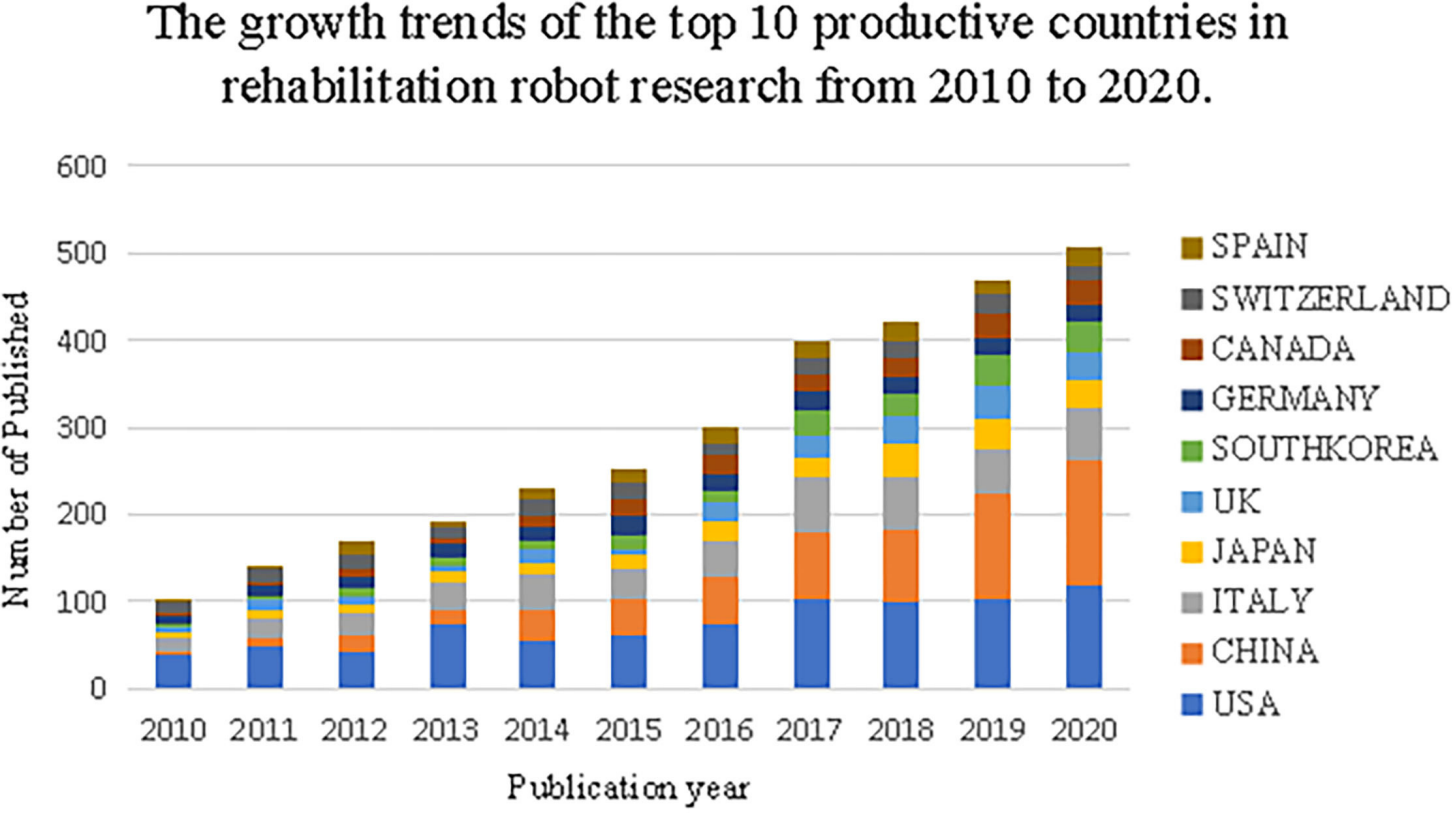
Bibliometric analysis of WoS core database output. From 2010 to 2020, the number of published publications by different countries on rehabilitation robot research has changed over the years.

A total of 3,194 publications met the inclusion criteria from 2010 to 2020 (Figure 1). Figure 2 shows the total number of publications annually, where the number of papers in rehabilitation robot research maintained a gradually increasing trend from 2010 to 2020.

### Hot Topics in Literature Research

**Figure 3:**
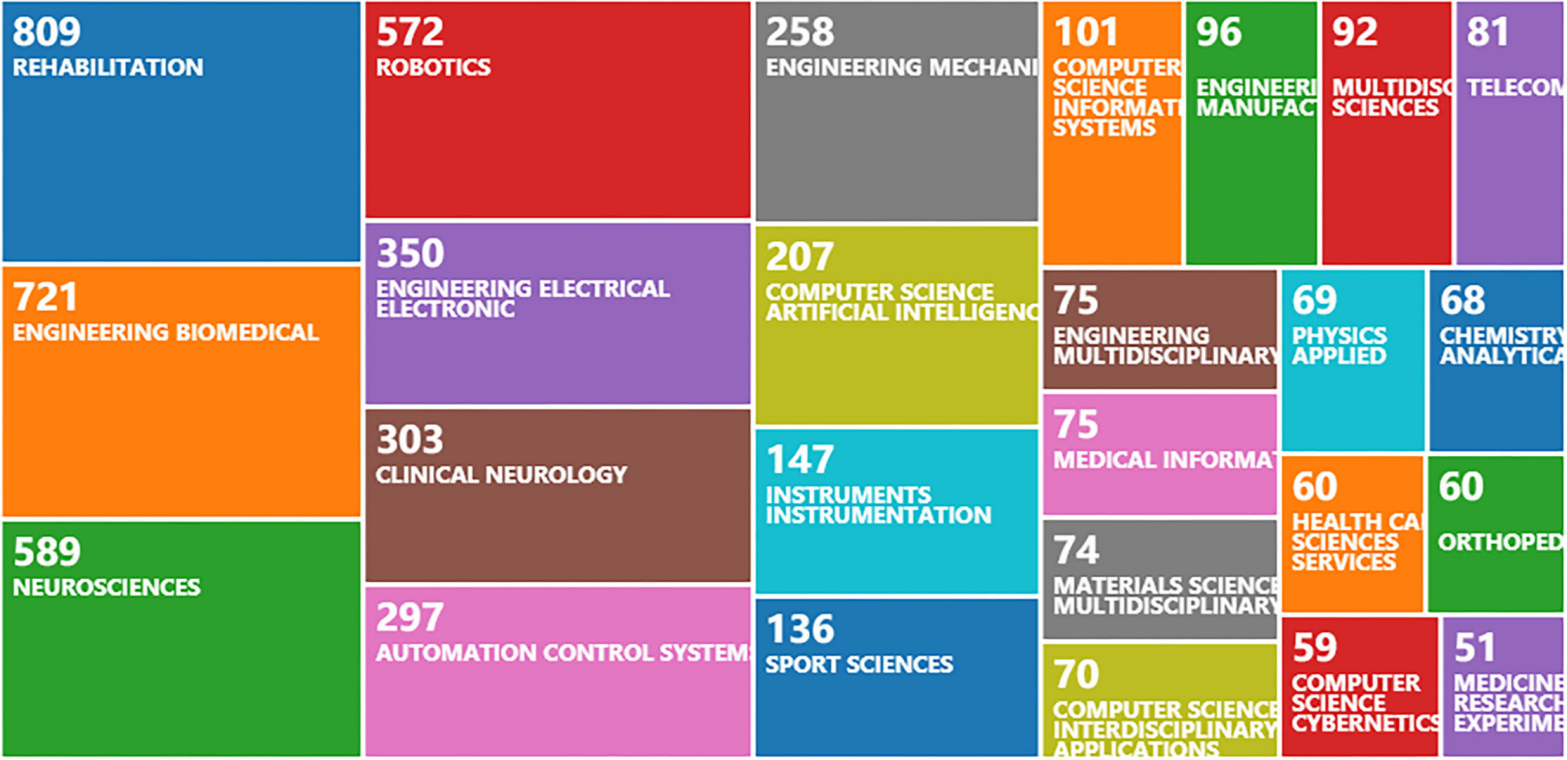
2010–2020 rehabilitation robot literature research hotspot distribution.

Among the 3,194 retrieved publications, according to the distribution analysis of literature research hotspots, the literature with more than 50 articles was classified into 25 categories (Figure 3). Of these, 809 belong to the category of rehabilitation medicine, 721 biomedical engineering, and 589 neuroscience. It can be seen from the data that rehabilitation robots are widely studied and applied in the fields of rehabilitation medicine, biomedical engineering, and neuroscience.

### Country or Geographic Analysis

**Figure 4:**
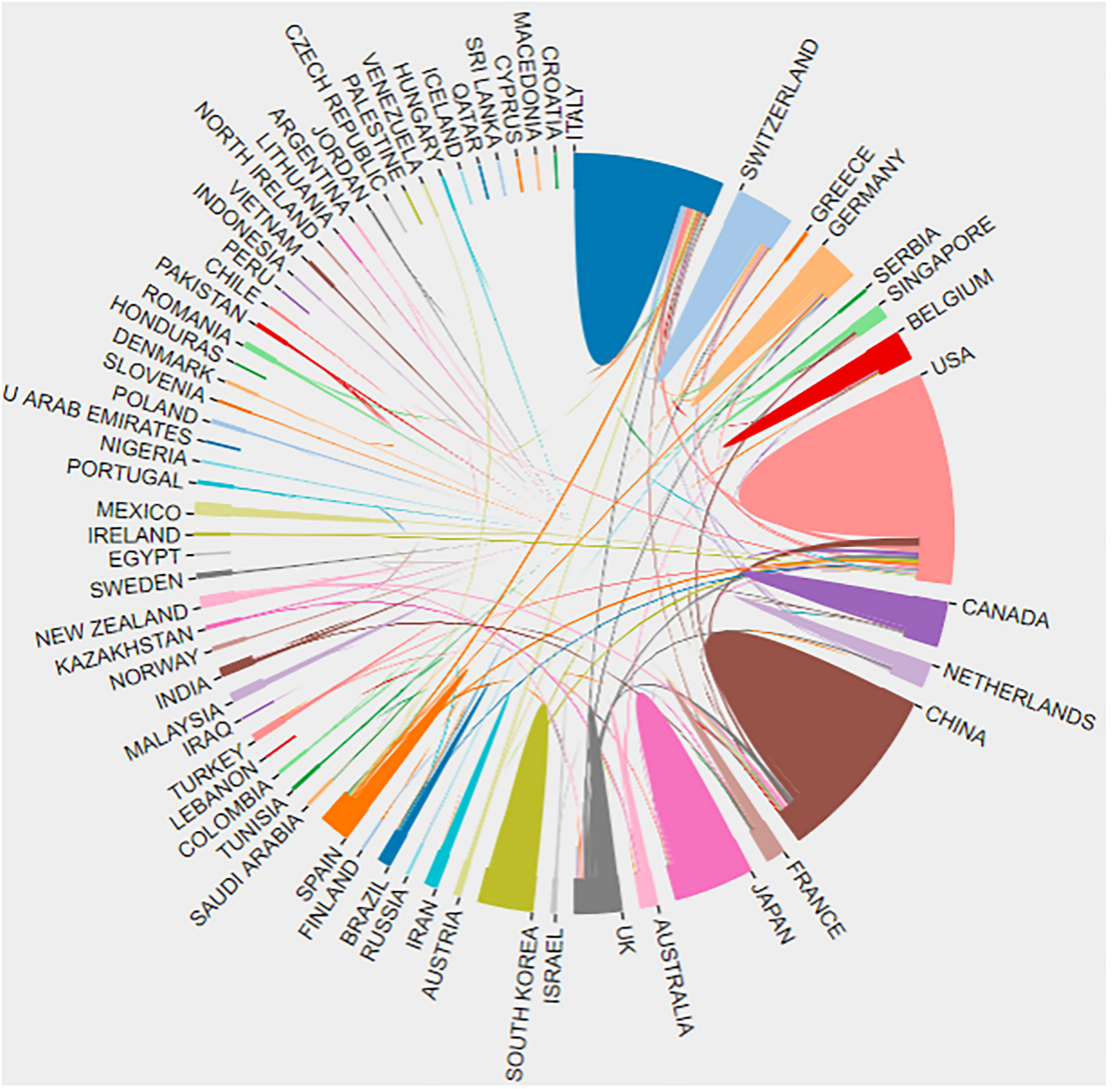
Bibliometric analysis of the cooperation of countries in the field of rehabilitation robot research.

A total of 82 countries or regions contributed to the research on rehabilitation robots, as shown in Table 1, among which the United States published the most articles (819). China ranked second with 603 publications. The third was Italy, with 444. A total of 2,605 institutions have published research related to rehabilitation robotics, the most prolific of which is Northwestern University (USA), with 161 publications. The second is the University of Tsukuba (Japan), with 133 publications. The University of Maryland ranked third with 81 publications (Table 1). Visualization of the study countries shows that the top three countries in centrality are Spain (0.4), the USA (0.26), and Germany (0.17). These three countries have established good research cooperation with other countries. The detailed cooperation between countries or regions is shown in Figure 4.

**Table 1:** Influence of high-yield countries and institutions on rehabilitation robot research literature from 2010 to 2020.

**Ranking**	**Countries/regions**	**No. of publications**	**Centrality**	**Institutions**	**No. of publications**
1	USA	819	0.26	Northwestern Univ	161
2	China	603	0.08	Univ Tsukuba	133
3	Italy	444	0.15	Univ Maryland	81
4	Japan	221	0.06	Scuola Super Sant Anna	81
5	UK	208	0.15	Univ Auckland	80
6	South Korea	187	0.03	Chang Gung Univ	80
7	Germany	181	0.17	Natl Univ Singapore	79
8	Canada	180	0.15	MIT	76
9	Switzerland	177	0.07	Columbia Univ	75
10	Spain	159	0.4	Univ Twente	61

### Research Hotspots Based on Cooccurrence of Research Institutions

**Figure 5:**
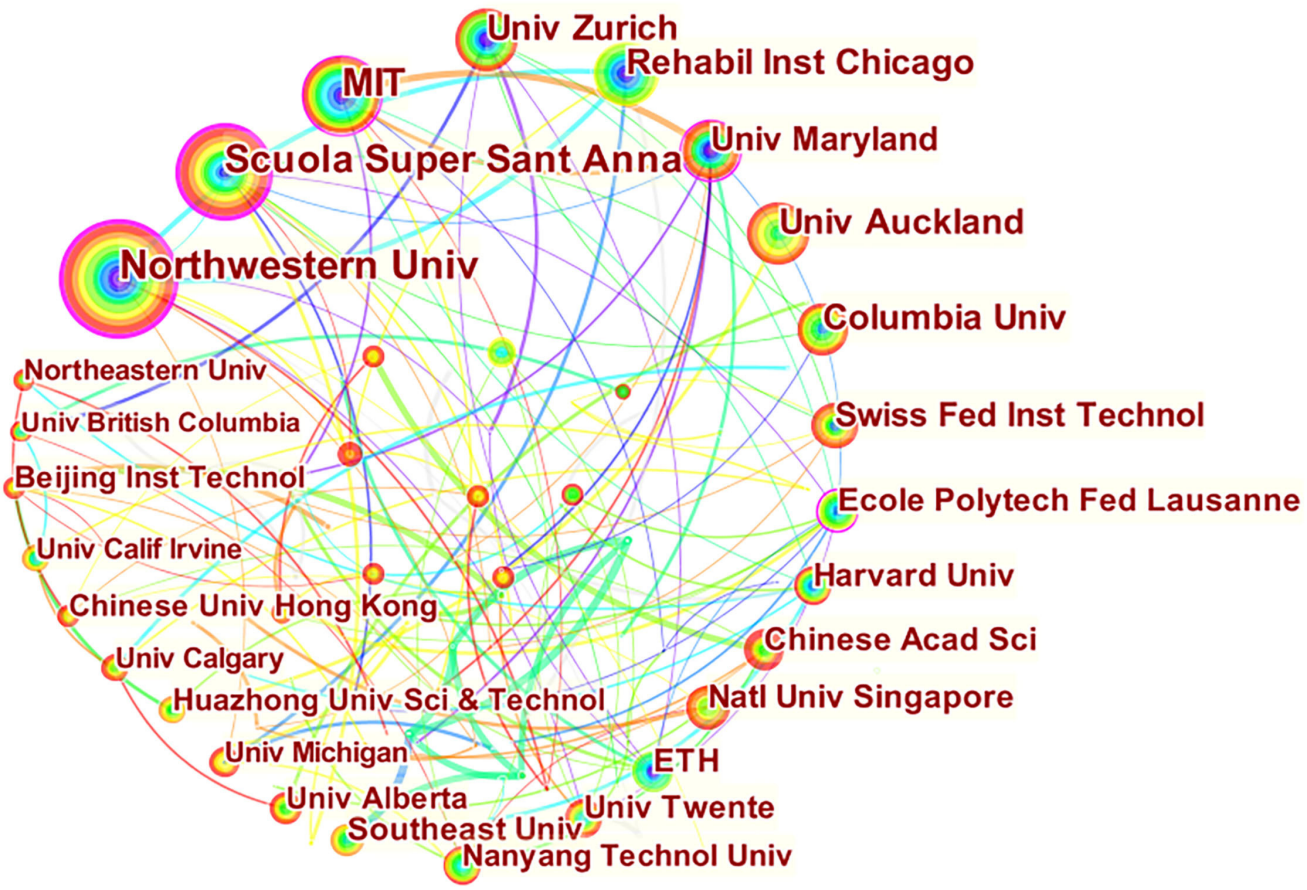
Co-occurrence atlas of rehabilitation robot research institutions. The bigger the circle, the more it appears. Lines refer to the connections between institutions. The thicker the lines, the closer the connections.

Through the analysis of cooperative cooccurrence of research institutions, CiteSpace software was used to draw the view of research hotspots of cooperative cooccurrence of research institutions of rehabilitation robots. A total of 411 nodes and 616 connections were generated, and the density of the topological network was 0.0073. Visualization of research institutions shows that Scuola Super Sant Anna (0.24), Northwestern University (0.23), and MIT (0.12) rank in the top three in centrality, respectively. These three institutions have established good research cooperation with other institutions (Figure 5).

### High-Influence Authors and Cooperative Relationships

**Figure 6:**
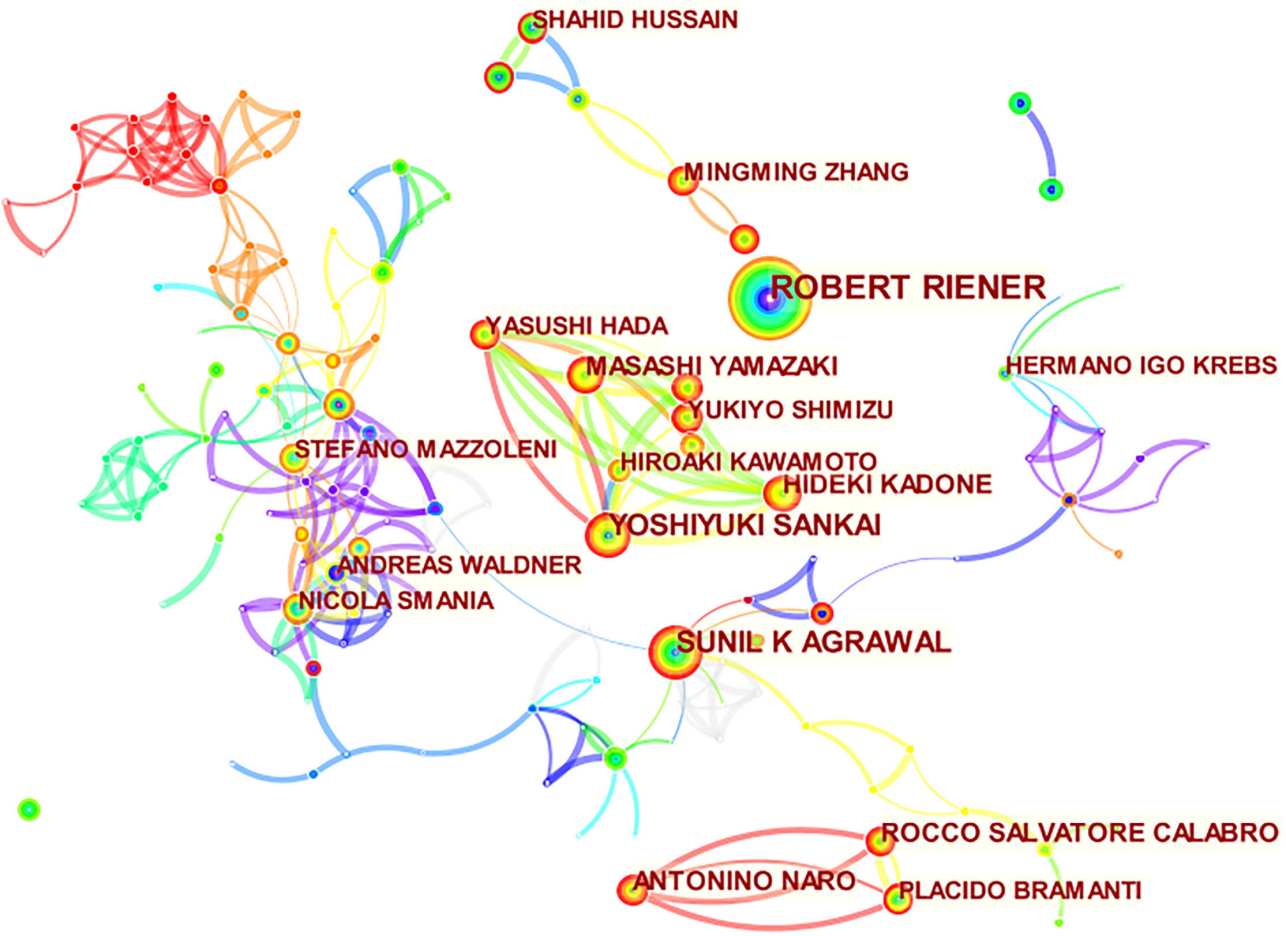
Author and cooperative relationship. The bigger the circle, the more it appears. Lines refer to the connections between authors. The thicker the lines, the closer the connections.

R. Riener, a professor at the University of Zurich in Switzerland, is the most prolific author with 48 articles, followed by S.K. Agrawal, a professor at Columbia University in the US, and Y. Sankai, a professor at the University of Tsukuba in Japan, with 29 articles. G. Kwakkel, a professor at the University of Amsterdam (512 times), followed by S. Hesse, a professor at Freie Universitat Berlin (512 times), and J. Mehrholz, a professor at Technische Universitat Dresden (335 times) (Table 2). In the author collaboration atlas, Professor R. Riener holds the leading position in this field by absolute advantage. However, there is no cooperative relationship with other authors with a high number of publications, and the cooperative relationship between other highly influential authors is also average (Figure 6).

**Table 2:** Status of authors with high influence on rehabilitation robot research from 2010 to 2020.

**Ranking**	**Highly prolific authors**	**Country**	**No. of publications**	**Highly cited authors**	**Total cites**	**Country**
1	Riener, R	Switzerland	48	Kwakkel, G	512	Netherlands
2	Agrawal, SK	USA	33	Hesse, S	438	Germany
3	Sankai, Y	Japan	29	Mehrholz, J	426	Germany
4	Kadone, H	Japan	23	Krebs, HI	393	USA
5	Yamazaki, M	Japan	23	Hogan, N	320	USA
6	Calabro, RS	Italy	23	Riener, R	308	Switzerland
7	Naro, A	Italy	21	Lo, AC	306	USA
8	Song A	China	18	Lum, PS	302	USA
9	Hada, Y	Japan	18	Veneman, JF	275	Spain
10	Micera, S	Switzerland	17	Reinkensmeyer, DJ	268	USA

### High-Influence Journals

Among the 3,194 articles retrieved, there are 515 source journals and the top 10 journals in terms of publication volume (Table 3). The top three journals in terms of publications are J Neuroeng Rehabil, IEEE T Neur Sys Reh, and IEEE Robot Autom Let.

**Table 3:** Status of high-impact journals on rehabilitation robot research from 2010 to 2020.

**Ranking**	**Journals**	**IF (2020)**	**JCR (2020)**	**No. of publications**	**Total cites**	**Average total cites**
1	J Neuroeng Rehabil	4.262	Q1	211	1,174	5.56
2	IEEE T Neur Sys Reh	3.800	Q1	167	821	4.92
3	IEEE Robot Autom Let	3.743	Q2	69	110	1.59
4	Sensors	3.574	Q1	66	65	0.98
5	IEEE Access	3.745	Q2	64	36	0.56
6	IEEE-ASME T Mech	5.673	Q1	59	488	8.27
7	Neurorehabilitation	2.134	Q2	55	200	3.64
8	Neurorehab Neural RE	3.914	Q1	54	603	11.17
9	Front Neurorobotics	2.650	Q2	51	72	1.41
10	Arch Phys Med Rehab	3.964	Q1	48	217	4.52

### Highly Cited Literature

Among the 3,194 retrieved articles, the total citation frequency was 47,224, and the top 10 cited (32–41) kinds of literature with high citation frequency (Table 4). The top three sources are LANCET, NEJM, and ROBOT AUTON SYST.

**Table 4:** References with high citation frequency of rehabilitation robots from 2010 to 2020.

**Ranking**	**Title**	**Publication year**	**Total cites**	**Journals**	**IF (2020)**	**JCR (2020)**
1	Stroke Care 2 stroke rehabilitation (20)	2011	952	Lancet	79.320	Q1
2	Robot-assisted therapy for long-term upper-limb impairment after stroke (21)	2010	704	NEJM	91.240	Q1
3	Soft robotic glove for combined assistance and at-home rehabilitation (22)	2014	485	Robot Auton Syst	3.123	Q1
4	The ReWalk powered exoskeleton to restore ambulatory function to individuals with thoracic-level motor-complete spinal cord injury (23)	2012	346	Am J Phys Med Rehab	2.152	Q2
5	Getting neurorehabilitation right: what can be learned from animal models? (24)	2012	281	Neurorehab Neural RE	3.914	Q1
6	Tactile-direction-sensitive and stretchable electronic skins based on human-skin-inspired interlocked microstructures (25)	2014	279	ACS Nano	15.884	Q1
7	An EMG-based control for an upper-limb power-assist exoskeleton robot (26)	2012	249	IEEE T Syst Man Cy B	13.450	Q1
8	Three-dimensional, task-specific robot therapy of the arm after stroke: a multicentre, parallel-group randomized trial (27)	2014	237	Lancet Neurol	44.182	Q1
9	Design and control of a bio-inspired soft wearable robotic device for ankle-foot rehabilitation (28)	2014	213	Bioinspir Biomim	2.952	Q1
10	Current hand exoskeleton technologies for rehabilitation and assistive engineering (29)	2012	212	Int J Precis Eng Man	2.104	Q2

### Research Hotspots Based on Keyword Cooccurrence

**Figure 7:**
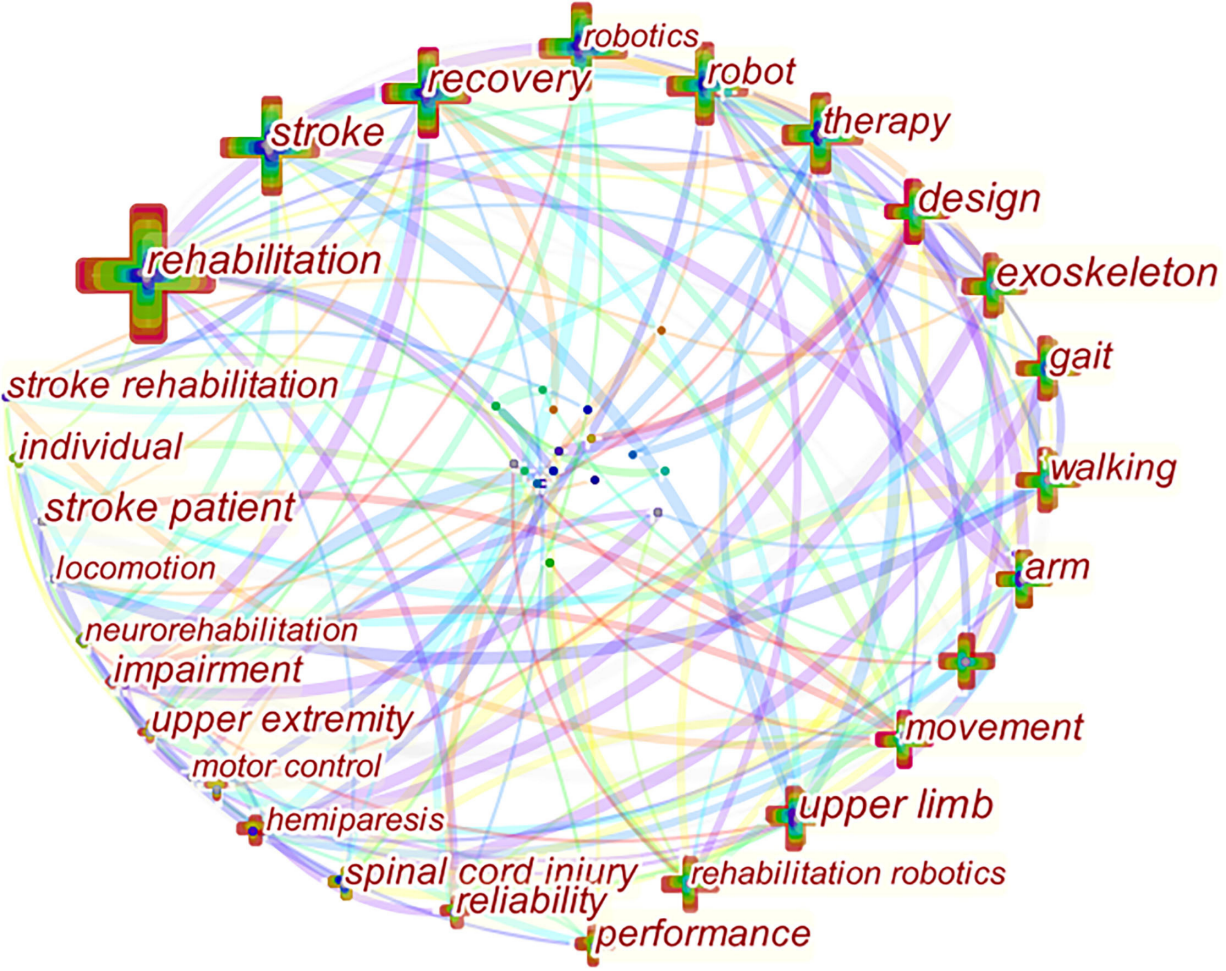
Co-occurrence spectrum of high-frequency keywords in rehabilitation robot field. The cross represents keywords; the larger the cross, the thicker the cross, the higher the frequency of keywords; the line refers to the connection between keywords; the thicker the line, the closer the connection.

Through keyword cooccurrence analysis, 20 high-frequency keywords with a frequency greater than 150 times were statistically ranked. The top three high-frequency keywords were “rehabilitation” (1,276 times), “stroke” (940 times), and “design” (471 times). The top three high-frequency keywords in centrality are “robot” (0.15), “walking” (0.15), and “movement” (0.15) (Table 5). CiteSpace software was used to draw the hotspot view of rehabilitation robot research. A total of 607 nodes and 2,728 connections were generated, and the density of the topological network was 0.0148 (Figure 7).

**Table 5:** High-frequency keywords of rehabilitation robot research from 2010 to 2020 (frequency > 150).

**Ranking**	**Keywords**	**Frequency**	**Centrality**	**Ranking**	**Keywords**	**Frequency**	**Centrality**
1	Rehabilitation	1,276	0.14	11	Gait	309	0.09
2	Stroke	940	0.13	12	System	289	0.06
3	Design	471	0.1	13	Arm	274	0.06
4	Recovery	469	0.12	14	Upper limb	256	0.11
5	Exoskeleton	434	0.11	15	Movement	238	0.15
6	Robot	418	0.15	16	Spinal cord injury	217	0.08
7	Therapy	373	0.1	17	Performance	198	0.15
8	Walking	345	0.15	18	Reliability	189	0.08
9	Rehabilitation robotics	326	0.11	19	Impairment	167	0.09
10	Robotics	316	0.06	20	Upper extremity	163	0.05

### Keywords Clustering Research Hotspots and Frontier

**Figure 8:**
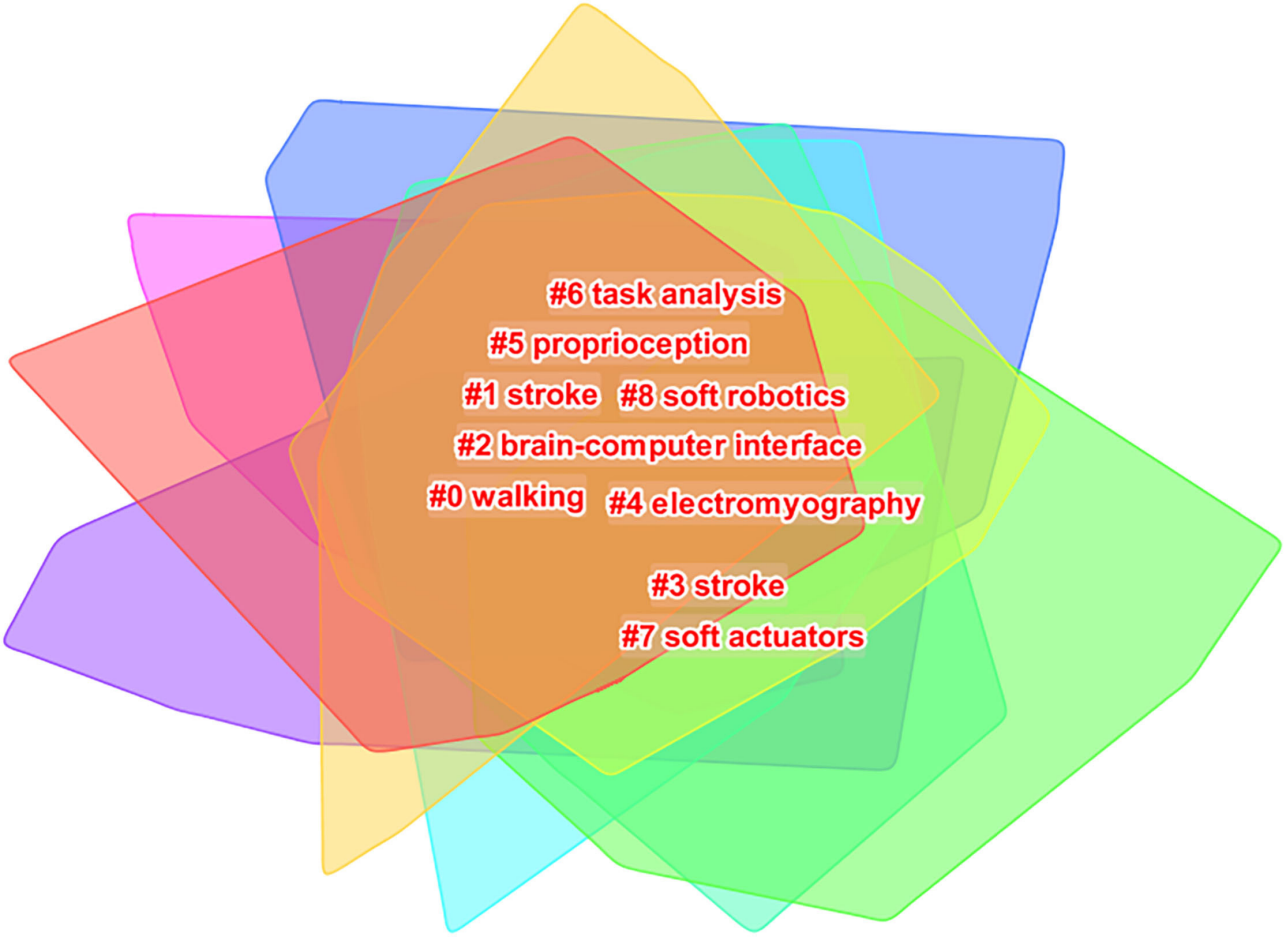
Cited cluster analysis showing the nine main research areas.

**Figure 9:**
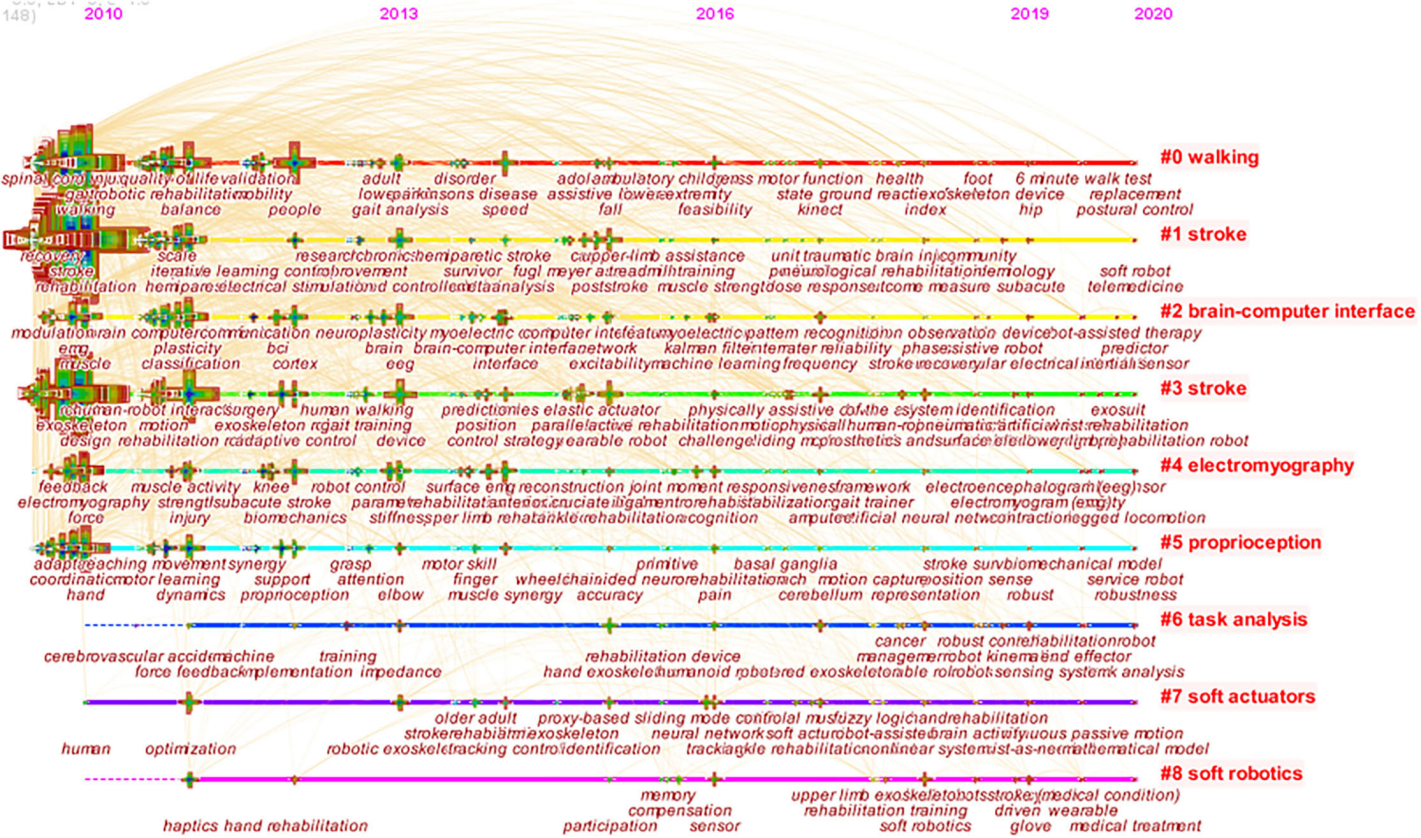
Time dynamic evolution of keywords.

CiteSpace was used for clustering analysis of keywords, and the classic LLR algorithm was adopted to obtain nine clustering groups: (0) walking, (1) stroke, (2) brain–computer interface, (3) stroke, (4) electromyography, (5) proprioception, (6) task analysis, (7) soft actuators, and (8) soft robotics (Figure 8). The timeline view is used to display the dynamic time change of clustering keywords. From 2010 rehabilitation robots, cerebral apoplexy, exoskeleton, the stability of the robot, rehabilitation, spinal cord injury, stroke, nerve rehabilitation, motor learning, virtual reality, and other keywords widely attention, among them, the rehabilitation robot and cerebrovascular accident, exoskeleton robot hand, adaptive control, and touch the heart of the study on the relationship between the recovery began in 2011. Rehabilitation robot and brain–computer interface, upper limb rehabilitation, and meta-analysis research began to appear in 2014. Research on the relationship between rehabilitation robots and upper limb rehabilitation and meta-analysis was hot until 2019. In 2019–2020, robot sensing system, actuator, pneumatic artificial muscle, telemedicine, task analysis, wearable robot, and flexible robot were new words. It is predicted that further research will focus on rehabilitation robots and robot sensing systems, pneumatic artificial muscle, wearable robot, and flexible robot. The time dynamic evolution of keywords is presented in Figure 9.

### Cocitation Clustering Analysis of Related Kinds of Literature

Through literature cocitation cluster analysis, CiteSpace software was used to draw a cocitation map of the rehabilitation robot research literature, generating 868 nodes and 1,884 connections, and the density of the topological network was 0.005. With the lowest citation times of 15, 28 of the 3,194 cited kinds of literature were included from the research scope, and the top three cited kinds of literature with high centrality were “Lo et al. (33)” (0.88), “Maciejasz et al. (42)” (0.68), and “Klamroth-Marganska et al. (39)” (0.45).

### Funding Support Institutions

There are 2,637 funded institutions, among which the top 10 funded institutions have funded 1,535 studies, accounting for 48% of the literature in the field of rehabilitation robot research. The top three funders were the China National Natural Science Research Foundation (337), the US Department of Health and Human Services (247), and the US National Institutes of Health (240). These results indicate that China has strong support for the research on rehabilitation robots and a large amount of research fund investment, which may be related to the incentive mechanism initiated by the government. However, there is still a gap between the total number and that of the United States, which needs to be improved (Table 6).

**Table 6:** Funding institutions of rehabilitation Robot Fund from 2010 to 2020.

**Ranking**	**Funding**	**No. of publications**	**Percentage**
1	National Natural Science Foundation of China NSFC	337	10.541
2	United States Department of Health Human Services	247	7.726
3	National Institutes of Health NIH USA	240	7.507
4	National Science Foundation NSF	162	5.067
5	NIH Eunice Kennedy Shriver National Institute of Child Health Human Development NICHD	117	3.66
6	European Union EU	111	3.472
7	Ministry of Education Culture Sports Science and Technology Japan MEXT	95	2.972
8	Japan Society for the Promotion of Science	86	2.69
9	Grants in AID for Scientific Research Kakenhi	72	2.252
10	NIH National Institute of Neurological Disorders Stroke NINDS	68	2.127

### Frontier Analysis of an Emergent Word Detection Algorithm

We used CiteSpace to detect emergent words. A total of 108 mutants were detected. Among the 25 keywords with the highest citation frequency, the mutation cycles of 16 mutation words were concentrated from 2010 to 2015. The strongest mutation intensity was “arm” (8), followed by “motor control” (7.13). The third place was “hemiparetic patient” (6.13), the fourth place was “body weight support” (5.14), and the fifth place was “arm movement” (5.11). Keywords with high mutation intensity in the last 5 years include “modulation” (2015–2016), “computer interface” (2015–2017), “treadmill therapy” (2016–2017), “series elastic actuator” (2017–2020), and “prosthetics and exoskeleton” “wearable robotics” (2018–2020). These emergent words in the past 5 years may also be the main direction of future research.

## Discussion

### International Research Status of Rehabilitation Robots

The research on rehabilitation robots can be traced back to the 1960s, but the development was relatively slow due to design and funding problems. The 1980s was the initial stage of the research on rehabilitation robots, and the United States and the United Kingdom were at the leading level. After the 1990s, the research on rehabilitation robots entered a period of comprehensive development (43). In 1987, the British man Michael developed the prototype of the Handy I rehabilitation robot. In 1989, the Massachusetts Institute of Technology developed the first clinical trial robot for rehabilitation treatment. In 2020, Elon Musk reported the latest research results on the brain–computer interface: a coin-sized chip implanted into a pig's brain can read the pig brain's information in real time. A functional device implanted into a surgical robot can perform all the steps of craniotomy, implant sensors, and glue. With the continuous development of interdisciplinary integration, researchers are making progress in the field of rehabilitation robots. It can be predicted that the vision of human–machine integration will be realized soon (44, 45).

### Research Hotspots by Country (Region), Author, and Founding

In this study, the research literature related to rehabilitation robots was obtained from the core data collection of the Web of Science database in the past 10 years as the research object, and CiteSpace visual analysis software was used to analyze and track the hotspots and frontiers of international research in the field of rehabilitation robots. This study presents the international research status of rehabilitation robots in the past 10 years intuitively and forms a general understanding of its research hotspots, frontiers, and trends. Overall, international rehabilitation robot research identified steady growth year by year, nerve rehabilitation, stroke, brain–machine interface, virtual reality, flexible wearables, task analysis, and the exoskeletons of rehabilitation robot research more and more aroused people's interests, becoming increasingly important areas of research.

The number of articles published in the world in the field of rehabilitation robots generally shows an upward trend, mainly focusing on the fields of rehabilitation medicine, biomedical engineering, and neuroscience, indicating that the research on rehabilitation robots is popular in the field of medicine. The United States, China, Italy, Japan, and the United Kingdom are the major research exporters. The United States has the most articles about rehabilitation robots, followed by China. China is the only developing country, which shows great progress in the field of rehabilitation robots in the past decade and playing an important role. In terms of research institutions, Northwestern University, the University of Tsukuba, the University of Maryland, Santa Anna University of Pisa, Italy, and the University of Auckland, New Zealand occupy a certain position in the field of rehabilitation robots with a high number of publications. Northwestern University ranked first with 161 publications.

Among the prolific authors, R. Riener, a professor at the University of Zurich in Switzerland, ranked first with 48 articles, and the second and third were from the United States and Japan, respectively. It is worth noting that Professor Song from Southeast University of China ranked eighth with 18 articles. Studies by Chinese scholars in the field of rehabilitation robots are moving toward the international community, even catching up abroad.

In terms of cocitation of authors, the top five are Prof G. Kwakkel, Vrije Universitat Amsterdam; Prof S. Hesse, Medical University of Charlotte, Berlin, Germany; Prof J. Mehrholz, Dresden Medical School, Technical University of Dresden, Germany; Prof H.I. Krebs, MIT, USA; and Prof N. Hogan, MIT, USA. It can be seen that scholars from the Netherlands, Germany, and the United States have high influence in the research on rehabilitation robots. The above data can provide potential collaborators and teams' information for future research. According to Price's law, half of all papers on the same subject are written by a group of highly productive authors roughly equal to the square root of the total number of authors (46). A total of 9,163 authors were included in 3,194 publications. Of these, the first 93 authors completed 50% of the papers, in line with Price's law. It indicates that the core authors in the field of rehabilitation robots have been established. Paying attention to these authors can help us better understand the frontier and trend of rehabilitation robots. Among the top five foundations, one is from China, and four are from the United States. The research area involves many scientific research grants, but the main funding areas are natural sciences, medicine, bioengineering, and material science.

### Research Hotspots in the Field of Rehabilitation Robots

Citation status is one of the important indicators to reflect research hotspots. Through reading and analyzing the literature with high citation frequency and centrality, some research results with high attention in the research field of rehabilitation robots can be learned to reveal the areas of concern (47). Through reading and analysis of the top ten cited studies, the results show that stroke, spinal cord injury, paraplegia, upper limb functional rehabilitation, neuroplasticity, actuator technology, flexible robot gloves, etc., have received extensive attention from researchers in a certain stage in the research field of rehabilitation robots. The research hotspots of these highly cited papers help to identify the major hotspots of rehabilitation robot research. At present, the number of cited papers in China is low, which may be due to the late publication of many studies in other countries.

Keywords with high centrality and frequency represent research hotspots in a period. According to the analysis of the hot keywords, research in the field of rehabilitation robots mainly focuses on rehabilitation, stroke, design, exoskeleton robotics, upper limbs, gait, spinal cord injury, performance, and reliability. Through keyword clustering analysis, the higher the degree of aggregation, the better the homogeneity between studies (48).

In the last 10 years, the rehabilitation of upper limb function for stroke has been a research hotspot. With the rapid growth of new technologies and equipment, there is much different training equipment for rehabilitation robots designed to move or assist arm movement. There are significant differences in the types of treatment provided by different devices in terms of the techniques employed and the treatments provided. In a systematic review, there is increasing evidence that rehabilitation robot training can benefit the recovery of arm function after stroke (49). Human–robot interaction (HRI) is one of the hot topics in the current research on rehabilitation robots, which is how to realize the natural and precise interaction between humans and machines through the fusion of machine intelligence and biological intelligence.

### Research Frontiers and Trends in Rehabilitation Robots

Emergent words represent frequently cited keywords in a given period, thus indicating cutting-edge features and trends. Combined with the time dynamic evolution and burst analysis of keywords, we can get a general understanding of the research field, development, and future trend of rehabilitation robots. The top five post-2014 breakout words are “modulation,” “computer interface,” “treadmill therapy,” “series elastic actuator,” and “prosthetics and exoskeleton.” Modulation refers to the adjustment and control of the signal and program of the rehabilitation robot to achieve the purpose of precise rehabilitation. Computer interface refers to the popular brain–computer interface technology. Treadmill therapy mainly refers to lower limb rehabilitation robots that are similar to treatments done on a treadmill. A Lokomat lower limb rehabilitation robot is a typical hanging weight loss standing rehabilitation robot of the treadmill therapy type, which combines with the treadmill platform to complete the exercise (50). In the control system, the series elastic actuator can convert control signals into power devices that can drive the controlled system, which is the core of the power development and design of the rehabilitation robot (51). Research on prosthetics and exoskeleton robots is currently a hot topic in comparison, and because of the rapid development of mechanical design and control algorithms for electro-mechanical systems, exoskeletons have been significantly developed but are still limited to larger body areas, such as the upper and lower limbs. Due to their small size and strong tactile perception, hand exoskeletons still face many challenges in many technical fields, such as hand biomechanics, neurophysiology, rehabilitation, actuators and sensors, human–robot physical interaction, and ergonomics.

In addition, machine learning (ML) is one of the technologies that have developed rapidly in the past 10 years. It is widely used in computer vision, bioinformatics, health care, business analysis, trend forecasting, and other fields. ML allows computers to learn from large samples of data and predict patterns that exist in the data (52, 53). ML algorithms to help realize the future of improved health care by unleashing the potential of large biomedical and patient data sets are used in different research areas to predict and classify accurate results from test data (54). They have proven to be helpful for diagnosis in a variety of medical and clinical data sets. In the field of rehabilitation, ML can help predict the recovery function of walking level of the patients with stroke and assist in research on the development of walking assistance robots. AI-based virtual reality rehabilitation (55) and forecasting mortality of patients with stroke after complete rehabilitation with tree-based ML models have been studied (56). For any type of human disease prediction, data sets need to be preprocessed. In this regard, dimensionality reduction plays a vital role in reducing the high-dimensional data into reduced dimensionality (57). Over the past few decades, several dimensionality reduction techniques have been used to filter data samples from the data set under consideration. Dimensionality reduction requires the mapping of higher-dimensional inputs to lower-dimensional inputs so that similar points in the input space are mapped to adjacent points on the manifold (54, 58). Feature engineering is an important preprocessing step that helps in the extraction of transformed features from the raw data that will simplify the ML model and also improve the quality of the results of an ML algorithm.

In the field of medical rehabilitation, more and more people tend to interact with machines naturally and accurately. With personalized AI task analysis, ML, electromechanical interaction, intelligent control, and other technologies combined with robots applied to the field of rehabilitation medicine, the rehabilitation robot “therapist” will become the main force in the field of rehabilitation therapy in future. The wearable exoskeleton robot can help elderly people with severe limb dysfunction, hemiplegia, and spinal cord injuries walk normally. In addition, it can help humans maintain balance function and increase strength to achieve “human–machine integration” (5). The “mind control” of brain–computer interface is gradually switching from science fiction to reality, helping the movement, feeling, and other functions of damaged limbs return to normal. In 2020, *MIT Technology Review* published a breakthrough in the brain–computer interface by Professor Eduardo Fernandez of Miguel Hernandez de Elche University: the electronic artificial eye, which restores sight to patients who are completely blind (59, 60).

With human intelligence and machine intelligence, combining human–computer interaction as a representative of technological breakthroughs between man and machine combined with the increasingly close, with the help of human–computer interaction technology and method, and combining human intelligence and machine intelligence make the two complementary advantages, to work together, and in the aspect of medical rehabilitation which spawned a significant theoretical innovation and breakthrough technology method. The brain–computer interface will be widely applied in future (61, 62). How to establish the ability of high-broadband data connection between human and machine is also an important trend in the current international rehabilitation robot research. At present, rehabilitation robots of limb function still cannot provide intuitive tactile neurofeedback, and patients can only rely on visual feedback to judge the size of the object to be grasped and other sensory information. How to achieve accurate neural feedback of sensory information still needs further research. It is the challenge and trend of the development of rehabilitation robots in future to make that rehabilitation robots have emotional characteristics and make patients feel happy while helping patients to achieve their rehabilitation goals.

Regarding rehabilitation robot technology and the research and development and application of the system, there is still a gap between China and developed countries such as Europe and the United States. To improve the performance of rehabilitation robot system, it is necessary to master the key core technologies of rehabilitation robot, such as EMG information perception and control technology, force feedback control technology, joint angle and torque control technology, and space motion detection technology (4). In particular, the research on these technologies in brain–computer interface, virtual reality, robot sensing system, actuators, pneumatic artificial muscle, and the wearable flexible robot can better promote the development of rehabilitation robots to meet the future demand of Chinese people for health services.

### Application and Development Trend of Rehabilitation Robots

It can be predicted that rehabilitation robots will be used more and more in an era when the cost of manual treatment is becoming more and more expensive. To better popularize and commercialize the application, future development needs to increase the universality of rehabilitation robots, focusing on lightweight, portable, reconfigurable, intelligent, and AI-based equipment, thus bringing new treatment methods and thinking to the rehabilitation field under the premise of ensuring safety, mass production, and cheap direction development (63). Intelligent rehabilitation is the future development trend (64). It should break through the barrier of the human–machine interface, develop multidisciplinary cooperation and communication, strengthen collaboration with rehabilitation medicine and artificial intelligence, actively participate in the design and development of rehabilitation robots, and promote rehabilitation robots to better serve the rehabilitation medicine field.

When thinking about the application and development of rehabilitation robots, Professor Li first mentioned the “human nature” of rehabilitation robots. Rehabilitation robots should have the basic human nature—namely, perception, thinking, expression and communication, action and cooperation, and learning and adaptability—and complete training and operations independently or with the assistance of an automatic machine (14). At present, rehabilitation robots cannot complete all the techniques and operations of professional rehabilitation personnel; particularly, the self-learning ability, adaptability, and creativity of robots need theoretical breakthroughs. The most difficult thing for rehabilitation robots to break through is the benevolence and moral sense of medical workers (65). The twenty first century is an era of health and life science. Health is the most powerful driving force for scientific and technological revolution and social development. Rehabilitation robots as one of the most important technologies in the human health industry will undoubtedly provide innovative means and paths for rehabilitation medicine, preventive medicine, and clinical medicine, making the previously invisible visible and the impossible possible (66). Rehabilitation robots will also provide strong impetus for the reform of the medical system, the renewal of the medical model, and the extension of the human healthy life span (67).

### Study Limitations

This study included only studies on rehabilitation robots published in the core data collection of SCIE, the Web of Science database, and the paper type was English literature, which may have ignored high-quality kinds of literature in other languages in the field of rehabilitation robots, resulting in certain limitations in literature retrieval. In cluster analysis, different categories of content may overlap. In addition, the cocitation frequency of kinds of literature is related to time. The kinds of literature published in recent years may have a relatively low total cocitation frequency due to their short publication time, resulting in differences between research results and the actual situation. The cooccurrence of keywords is limited by the number of nodes, which may affect the accuracy of conclusions. However, accuracy itself is relative and does not affect the reliability of conclusions obtained in the paper. Due to different algorithms, there is no standardized setting process for time partition, threshold, or clipping mode in the process of using CiteSpace to generate visual atlas, which may cause some bias.

## Conclusion

Based on the Web of Science database and CiteSpace software, this study for the first time conducted bibliometric and visual analysis of the international research on rehabilitation robots in the past 10 years from multiple perspectives and presented the research overview of rehabilitation robots in the past 10 years relatively scientifically and intuitively. At present, the brain–computer interface, virtual reality, flexible wearables, task analysis, and exoskeleton rehabilitation robots are the frontiers and hotspots of research, representing the development trend of future research, and they can be used as a reference direction for future research. Future research in the field of rehabilitation robots should focus on the following aspects: first, focus on the functional needs of patients to improve the quality of life; second, strengthen interdisciplinary cooperation and build a regional cooperation network among countries.

### Challenges

Formulating better rehabilitation robots in such an evolving field is important. Until today, numerous technical challenges and insufficient safety standards have prevented the large-scale development of rehabilitation robots. As it is multidisciplinary technical research, it faces many challenges in the actual research and development process. The primary challenge of rehabilitation robots is safety, which is the most basic requirement to help patients with rehabilitation training. On the premise of ensuring the effectiveness of the product, it is more important to ensure the safety of use and avoid secondary injuries to patients. Intelligent control, ergonomics, at present, and the movement energy consumption of the robot bionic man are much higher than that of the simple skeletal muscle movement, the operation process of the wearable exoskeleton robot is generally more complex, and the large weight makes the rehabilitation training more difficult, but the precision is much worse. The man–machine interface design is a challenging problem. This interface is the medium and dialogue window for transmitting and exchanging information between humans and computers. A good man–machine interface is key to improve the operation efficiency of rehabilitation robots. For example, patients' subjective feelings and physiological reactions, especially visual perception, cannot be timely perceived in the training process. Both need constant optimization and learning. Existing rehabilitation robots generally lack tactile feedback. It is suggested to study various sensors to enhance the interaction between rehabilitation robots and the human body, involving multidisciplinary cooperation and communication. In addition, a key challenge regarding the control of rehabilitation robots is to design shared control or ML technologies that are predictable to patients and do not exceed their needs. Because not all technical components of rehabilitation robots are well developed or designed for daily life and outdoor applications, a lot of collaborative work and utilization of resources from medical technology, biomechanics, engineering, and product development are needed. The price of the current rehabilitation robots is relatively expensive, which is a great burden on ordinary families. It is suggested that future research can find cost-effective materials to popularize rehabilitation robots in ordinary life.

### Future Direction

In terms of technology, ML, dimensionality reduction, feature engineering, and other technologies play an important role in the research and development of rehabilitation robots. In future, the effectiveness of these technologies can be tested on high-dimensional data such as images and text data. They can also be used for more complex algorithms, such as deep neural networks, convolutional neural networks, and recursive neural networks. The new ideas of mixing and matching hardware, programs, algorithms, and expected neural pathways may lead to more focused research and, ultimately, significant advances in robot-assisted rehabilitation. For various rehabilitation robots with different characteristics and advantages, the application effect of different rehabilitation programs is not clear, so it is particularly important to formulate the user guide of rehabilitation robots to direct the clinical application of rehabilitation robots. To achieve widespread application and commercialization, future rehabilitation robots should be developed toward mass production and low cost. In addition, in future, rehabilitation robots will still be the focus and hotspot in the fields of rehabilitation medicine and intelligent engineering, and with the development of rehabilitation robots with different functions, their usage guidelines will become more and more perfect, and the cooperation between rehabilitation therapists and rehabilitation robots is bounded to open a new chapter in rehabilitation therapy.

## Data Availability Statement

The data analyzed in this study is subject to the following licenses/restrictions: when used on a reasonable request, it can be obtained by contacting the corresponding author. Requests to access these datasets should be directed to lining@cdsu.edu.cn.

## Author Contributions

XX and XY: designing this study and writing initial draft and revision. ZD and HT: reviewing the literature and analyzing. DK: making figures and tables. FX: rechecking the manuscript and putting forward suggestions for amendment. NL: revising language and content, supervision, project administration, and funding acquisition. All authors contributed to the article and approved the submitted version.

## Funding

This work was supported by the Key Laboratory of Sports Medicine of Sichuan Province, Institute of Sports Medicine and Health, Chengdu Sport University (No. 2021-A030) and the National Key Research and Development Program of China (No. 2018YFF0300904).

## Conflict of Interest

The authors declare that the research was conducted in the absence of any commercial or financial relationships that could be construed as a potential conflict of interest.

## Publisher's Note

All claims expressed in this article are solely those of the authors and do not necessarily represent those of their affiliated organizations, or those of the publisher, the editors and the reviewers. Any product that may be evaluated in this article, or claim that may be made by its manufacturer, is not guaranteed or endorsed by the publisher.

## References

[1] Fiske A Henningsen P Buyx A. Your robot therapist will see you now: ethical implications of embodied artificial intelligence in psychiatry, psychology, and psychotherapy. J Med Internet Res. (2019) 21:e13216. 10.2196/13216 31094356 PMC6532335

[2] Akbari A Haghverd F Behbahani S. Robotic home-based rehabilitation systems design: from a literature review to a conceptual framework for community-based remote therapy during COVID-19 pandemic. Front Robot AI. (2021) 8:612331. 10.3389/frobt.2021.612331 34239898 PMC8258116

[3] Kaelin VC Valizadeh M Salgado Z Parde N Khetani MA. Artificial intelligence in rehabilitation targeting the participation of children and youth with disabilities: scoping review. J Med Internet Res. (2021) 23:e25745. 10.2196/25745 34734833 PMC8603165

[4] Li G Zheng Y Wu X Hu Y Fang P Xiong J Xia Z Wang C. Research progress and trend of medical rehabilitation robot. Proc Chin Acad Sci USA. (2015) 30:793–802. 10.16418/j.issn.1000-3045.2015.06.013

[5] Yu H. Rehabilitation robots: ten visions for the future. Chin J Rehabil Med. (2020) 35:900–2. 10.3969/j.issn.1001-1242.2020.08.002

[6] Wang Q Wei Y Liu L. Progress in research and application of rehabilitation robots. Pack Eng. (2018) 39:83–9. 10.19554/j.cnki.1001-3563.2018.18.018

[7] Li H Zhang T Feng Y Lin H Bai D. Application of exoskeleton-based lower limb rehabilitation robot in stroke rehabilitation. Chin Rehabil Theory Pract. (2017) 023(007):788-791. 10.3969/j.issn.1006-9771.2017.07.010

[8] Butchart J Harrison R Ritchie J Martí F McCarthy C Knight S. Child and parent perceptions of acceptability and therapeutic value of a socially assistive robot used during pediatric rehabilitation. Disabil Rehabil. (2021) 43:163–170. 10.1080/09638288.2019.1617357 31120794

[9] Fang J Schuwey A Stocker N Pedrini B Sampaio A Hunt KJ. Preliminary development and technical evaluation of a belt-actuated robotic rehabilitation platform. Technol Health Care. (2021) 29:595–607. 10.3233/THC-202392 32741796 PMC8203225

[10] Nam C Rong W Li W Cheung C Ngai W Cheung T. An exoneuromusculoskeleton for self-help upper limb rehabilitation after stroke. Soft Robot. (2020). 10.1089/soro.2020.0090 33271057 PMC8885439

[11] Pérez-San Lázaro R Salgado I Chairez I. Adaptive sliding-mode controller of a lower limb mobile exoskeleton for active rehabilitation. ISA Trans. (2021) 109:218–228. 10.1016/j.isatra.2020.10.008 33077173

[12] Rodgers H Bosomworth H Krebs HI van Wijck F Howel D Wilson N. Robot assisted training for the upper limb after stroke (RATULS): a multicentre randomised controlled trial. Lancet. (2019) 394:51–62. 10.1016/S0140-6736(19)31055-4 31128926 PMC6620612

[13] Bernhardt J Mehrholz J. Robotic-assisted training after stroke: RATULS advances science. Lancet. (2019) 394:6–8. 10.1016/S0140-6736(19)31156-0 31128923

[14] Li J. Human-machine integration, nature and man integration—thoughts on the application and development of rehabilitation robots. Chin J Rehabil Med. (2020) 35:897–9. 10.3969/j.issn.1001-1242.2020.08.001

[15] Mehrholz J Hädrich A Platz T Kugler J Pohl M. Electromechanical and robot-assisted arm training for improving generic activities of daily living, arm function, and arm muscle strength after stroke. Cochrane Database Syst Rev. (2012) 6:CD006876. 10.1002/14651858.CD006876.pub3 22696362

[16] Nam KY Kim HJ Kwon BS Park JW Lee HJ Yoo A. Robot-assisted gait training (Lokomat) improves walking function and activity in people with spinal cord injury: a systematic review. J Neuroeng Rehabil. (2017) 14:24. 10.1186/s12984-017-0232-3 28330471 PMC5363005

[17] Lefmann S Russo R Hillier S. The effectiveness of robotic-assisted gait training for paediatric gait disorders: systematic review. J Neuroeng Rehabil. (2017) 14:1. 10.1186/s12984-016-0214-x 28057016 PMC5217646

[18] Krebs HI Volpe BT. Rehabilitation robotics. Handbook Clin Neurol. (2013) 110:283–94. 10.1016/B978-0-444-52901-5.00023-X 23312648 PMC4688009

[19] Kim WS Cho S Ku J Kim Y Lee K Hwang HJ. Clinical application of virtual reality for upper limb motor rehabilitation in stroke: review of technologies and clinical evidence. J Clin Med. (2020) 9:3369. 10.3390/jcm9103369 33096678 PMC7590210

[20] Keeling AB Piitz M Semrau JA Hill MD Scott SH Dukelow SP. Robot enhanced stroke therapy optimizes rehabilitation (RESTORE): a pilot study. J Neuroeng Rehabil. (2021) 18:10. 10.1186/s12984-021-00804-8 33478563 PMC7819212

[21] Chien WT Chong YY Tse MK Chien CW Cheng HY. Robot-assisted therapy for upper-limb rehabilitation in subacute stroke patients: a systematic review and meta-analysis. Brain Behav. (2020) 10:e01742. 10.1002/brb3.1742 32592282 PMC7428503

[22] Ranzani R Lambercy O Metzger JC Califfi A Regazzi S Dinacci D. Neurocognitive robot-assisted rehabilitation of hand function: a randomized control trial on motor recovery in subacute stroke. J Neuroeng Rehabil. (2020) 17:115. 10.1186/s12984-020-00746-7 32831097 PMC7444058

[23] Synnestvedt MB Chen C Holmes JH. CiteSpace II: visualization and knowledge discovery in bibliographic databases. In: AMIA Annual Symposium Proceedings AMIA Symposium. (2005), p. 724–8. 16779135 PMC1560567

[24] Chen C Chen Y. Searching for clinical evidence in CiteSpace. In: AMIA Annual Symposium Proceedings AMIA Symposium. (2005), p. 121–5. 16779014 PMC1560638

[25] Chaomei C Zhigang H Shengbo L Hung T. Emerging trends in regenerative medicine: a scientometric analysis in CiteSpace. Expert Opin Biol Ther. (2013) 2012:593–608. 10.1517/14712598.2012.674507 22443895

[26] Sun W Huang P Song H Feng D. Bibliometric analysis of acute pancreatitis in Web of Science database based on CiteSpace software. Medicine. (2020) 99:e23208. 10.1097/MD.0000000000023208 33285696 PMC7717787

[27] Lu C Li X Yang K. Trends in shared decision-making studies from 2009 to 2018: a bibliometric analysis. Front. Public Health. (2019) 7:384. 10.3389/fpubh.2019.00384 31921749 PMC6930165

[28] van Hedel HJA Severini G Scarton A O'Brien A Reed T Gaebler-Spira D. Advanced Robotic Therapy Integrated Centers (ARTIC): an international collaboration facilitating the application of rehabilitation technologies. J Neuroeng Rehabil. (2018) 15:30. 10.1186/s12984-018-0378-7 29625628 PMC5889593

[29] Liang C Luo A Zhong Z. Knowledge mapping of medication literacy study: A visualized analysis using CiteSpace. SAGE Open Med. (2018) 6:2050312118800199. 10.1177/2050312118800199 30245817 PMC6144508

[30] Ping X. Study of international anticancer research trends via co-word and document co-citation visualization analysis. Scientometrics. (2015) 105:611–22. 10.1007/s11192-015-1689-0

[31] Xia D Yao R Wang S Chen G Wang Y. Mapping trends and hotspots regarding clinical research on COVID-19: a bibliometric analysis of global research. Front. Public Health. (2021) 9:713487. 10.3389/fpubh.2021.713487 34497794 PMC8419357

[32] Langhorne PP Bernhardt PJ Kwakkel G. Stroke care 2: stroke rehabilitation. Lancet. (2011) 377:1693–702. 10.1016/S0140-6736(11)60325-5 21571152

[33] Lo AC Guarino PD Richards LG Haselkorn JK Wittenberg GF Federman DG. Robot-assisted therapy for long-term upper-limb impairment after stroke. N Engl J Med. (2010) 362:1772–83. 10.1056/NEJMoa0911341 20400552 PMC5592692

[34] Polygerinos P Wang Z Galloway KC Wood RJ Walsh CJ. Soft robotic glove for combined assistance and at-home rehabilitation. Robot Autonom Syst. (2014) 73(C):135–43. 10.1016/j.robot.2014.08.014

[35] Esquenazi A Talaty M Packel A Saulino M. The ReWalk powered exoskeleton to restore ambulatory function to individuals with thoracic-level motor-complete spinal cord injury. Am J Phys Med Rehabil. (2012) 91:911–21. 10.1097/PHM.0b013e318269d9a3 23085703

[36] Krakauer JW Carmichael ST Corbett D Wittenberg GF. Getting neurorehabilitation right: what can be learned from animal models? Neurorehabil Neural Repair. (2012) 26:923. 10.1177/1545968312440745 22466792 PMC4554531

[37] Park J Lee Y Hong J Lee Y Ha M Jung Y. Tactile-direction-sensitive and stretchable electronic skins based on human-skin-inspired interlocked microstructures. ACS Nano. (2014) 8:12020–9. 10.1021/nn505953t 25389631

[38] Kiguchi K Hayashi Y. An EMG-based control for an upper-limb power-assist exoskeleton robot. IEEE Trans Syst Man Cybern Part B. (2012) 42:1064–71. 10.1109/TSMCB.2012.2185843 22334026

[39] Klamroth-Marganska V Blanco J Campen K Curt A Dietz V Ettlin T. Three-dimensional, task-specific robot therapy of the arm after stroke: a multicentre, parallel-group randomised trial. Lancet Neurol. (2014) 13:159–66. 10.1016/S1474-4422(13)70305-3 24382580

[40] Park YL Chen BR Pérez-Arancibia NO Young D Stirling L Wood RJ. Design and control of a bio-inspired soft wearable robotic device for ankle-foot rehabilitation. Bioinspir Biomimet. (2014) 9:016007. 10.1088/1748-3182/9/1/016007 24434598

[41] Heo P Gu GM Lee SJ Rhee K Kim J. Current hand exoskeleton technologies for rehabilitation and assistive engineering. Int J Precis Eng Manuf. (2012) 13:807–824. 10.1007/s12541-012-0107-228475207

[42] Maciejasz P Eschweiler J Gerlach-Hahn K Jansen-Troy A Leonhardt S. A survey on robotic devices for upper limb rehabilitation. J Neuroeng Rehabil. (2014) 11:1–29. 10.1186/1743-0003-11-3 24401110 PMC4029785

[43] Prior SD Warner PR. A review of world rehabilitation robotics research. In: IEEE Colloquium on High-tech Help for the Handicapped (2002).

[44] Musk E. An integrated brain-machine interface platform with thousands of channels. J Med Internet Res. (2019) 21:e16194. 10.2196/16194 31642810 PMC6914248

[45] Singh HR Mobin A Kumar S Chauhan S Agrawal SS. Design and development of voice/joystick operated microcontroller based intelligent motorised wheelchair. In: Tencon 99 IEEE Region 10 Conference (1999).

[46] Price DDS. Little science big science. Paperback. (1963)

[47] Ahmad P Asif JA Alam MK Slots J. A bibliometric analysis of Periodontology 2000. Periodontology. (2000) 82:286–297. 10.1111/prd.12328 31850637

[48] He J He L Geng B Xia Y. Bibliometric analysis of the top-cited articles on unicompartmental knee arthroplasty. J Arthroplasty. (2021) 36:1810–8.e3. 10.1016/j.arth.2020.11.038 33423879

[49] Mehrholz J Pollock A Pohl M Kugler J Elsner B. Systematic review with network meta-analysis of randomized controlled trials of robotic-assisted arm training for improving activities of daily living and upper limb function after stroke. J Neuroeng Rehabil. (2020) 17:83. 10.1186/s12984-020-00715-0 32605587 PMC7325016

[50] Schicketmueller A Lamprecht J Hofmann M Sailer M Rose G. Gait event detection for stroke patients during robot-assisted gait training. Sensors. (2020) 20:3399. 10.3390/s20123399 32560256 PMC7349052

[51] Blaya JA Herr H. Adaptive control of a variable-impedance ankle-foot orthosis to assist drop-foot gait. IEEE Trans Neural Syst Rehabil Eng. (2004) 12:24–31. 10.1109/TNSRE.2003.823266 15068184

[52] Thippa RG Praveen K Lakshmanna K Kaluri R Baker T. Analysis of dimensionality reduction techniques on big data. IEEE Access. (2020) 8:54776–88. 10.1109/ACCESS.2020.298094227295638

[53] Sarhan MH Nasseri MA Zapp D Maier M Lohmann CP Navab N. Machine learning techniques for ophthalmic data processing: a review. IEEE J Biomed Health Inform. (2020) 24:3338–50. 10.1109/JBHI.2020.3012134 32750971

[54] Liu Y Zhang R Nie F Li X Ding C. Supervised dimensionality reduction methods via recursive regression. IEEE Trans Neural Netw Learning Syst. (2020) 31:3269–79. 10.1109/TNNLS.2019.2940088 31603803

[55] Seo K Chung B Panchaseelan HP Kim T Park H Oh B. Forecasting the walking assistance rehabilitation level of stroke patients using artificial intelligence. Diagnostics. (2021) 11:1096. 10.3390/diagnostics11061096 34204000 PMC8232707

[56] Scrutinio D Ricciardi C Donisi L Losavio E Battista P Guida P. Machine learning to predict mortality after rehabilitation among patients with severe stroke. Sci Rep. (2020) 10:20127. 10.1038/s41598-020-77243-3 33208913 PMC7674405

[57] Becht E McInnes L Healy J Dutertre CA Kwok IWH Ng LG. Dimensionality reduction for visualizing single-cell data using UMAP. Nat Biotechnol. (2018) 374:20150202. 10.1038/nbt.4314 30531897

[58] Sun X Liu Y An L. Ensemble dimensionality reduction and feature gene extraction for single-cell RNA-seq data. Nat Commun. (2020) 11:5853. 10.1038/s41467-020-19465-7 33203837 PMC7673125

[59] Page RJ. A New Implant for Blind People Jacks Directly into the Brain. MIT Technology Review. (2020). Available online at: https://www.technologyreview.com/2020/02/06/844908/a-new-implant-for-blind-people-jacks-directly-into-the-brain/ (accessed February 6, 2020).

[60] Zhang X Chan FK Parthasarathy T Gazzola M. Modeling and simulation of complex dynamic musculoskeletal architectures. Nat Commun. (2019) 10:4825. 10.1038/s41467-019-12759-5 31645555 PMC6811595

[61] Xue X Tu H Deng Z Zhou L Li N Wang X. Effects of brain-computer interface training on upper limb function recovery in stroke patients: a protocol for systematic review and meta-analysis. Medicine. (2021) 100:e26254. 10.1097/MD.0000000000026254 34115016 PMC8202595

[62] Abbruzzese G Marchese R Avanzino L Pelosin E. Rehabilitation for Parkinson's disease: current outlook and future challenges. Parkinsonism Relat Disord. (2016) 22 Suppl 1:S60–4. 10.1016/j.parkreldis.2015.09.005 26360239

[63] Khor KX Chin PJH Yeong CF Su ELM Narayanan ALT Abdul Rahman H. Portable and reconfigurable wrist robot improves hand function for post-stroke subjects. IEEE Trans Neural Syst Rehabil Eng. (2017) 25:1864–73. 10.1109/TNSRE.2017.2692520 28410110

[64] Lu L Zhang J Xie Y Gao F Xu S Wu X. Wearable health devices in health care: narrative systematic review. JMIR mHealth uHealth. (2020) 8:e18907. 10.2196/18907 33164904 PMC7683248

[65] Treviño LR Roberge P Auer ME Morales A Torres-Reveron A. Predictors of functional outcome in a cohort of hispanic patients using exoskeleton rehabilitation for cerebrovascular accidents and traumatic brain injury. Front Neurorobot. (2021) 15:682156. 10.3389/fnbot.2021.682156 34177511 PMC8222710

[66] Hussain S Jamwal PK Vliet PV Brown NAT. Robot assisted ankle neuro-rehabilitation: state of the art and future challenges. Exp Rev Neurotherap. (2021) 21:111–21. 10.1080/14737175.2021.1847646 33198522

[67] Klamroth-Marganska V. Stroke rehabilitation: therapy robots and assistive devices. Adv Exp Med Biol. (2018) 1065:579–87. 10.1007/978-3-319-77932-4_35 30051408

